# Evaluating data quality at a primary healthcare rehabilitation centre in a low-resource context

**DOI:** 10.1186/s12875-026-03290-w

**Published:** 2026-05-23

**Authors:** Maria Y. Charumbira, Gabriela B. Prins, Quinette A. Louw

**Affiliations:** Division of Physiotherapy, Department of Rehabilitation and Health Sciences, Faculty of Medicine and Health Sciences, Stellenbosch University, Cape Town, South Africa

**Keywords:** Data quality, Health information systems, Primary healthcare, Rehabilitation, South Africa

## Abstract

**Background:**

Reliable rehabilitation data are essential for planning, monitoring, and strengthening health systems. In low-resource primary healthcare settings, however, data quality remains a challenge. This study assessed the availability, completeness, and consistency of rehabilitation data at a primary healthcare rehabilitation centre in South Africa.

**Methods:**

A facility-based retrospective descriptive case study was conducted through a document review of annual reports and supporting documents from 2014 to 2023. The World Health Organization’s Guidance on Routine Health Information Systems – Rehabilitation module was used as the assessment standard to evaluate the completeness and consistency of the rehabilitation centre’s available data. Data were analysed descriptively to evaluate key data elements’ completeness and consistency across years and rehabilitation disciplines (physiotherapy, occupational therapy, and speech-language therapy).

**Results:**

Loss of data, staff turnover and lack of a centralized health information system affected the availability of data. Of the 9 core indicators assessed, none were fully complete; five were partially complete, four were entirely absent. Completeness of key data elements varied from 34 to 80%. Lack of data consistency was observed between disciplines and across reporting years, including non-standardized reporting formats, different naming or grouping of categories, and unexplained gaps and fluctuations in the data.

**Conclusion:**

The study highlights substantial gaps in the availability and quality of rehabilitation data at the primary healthcare facility. Findings underscore the need for standardized data collection tools, capacity-building in data management, and integration of rehabilitation indicators into electronic health information systems. Strengthening data quality mechanisms is critical not only for PHC rehabilitation services in South Africa but also for other low-resource settings seeking to advance evidence-based rehabilitation policy, planning, and research.

**Trial registration:**

Not applicable.

## Introduction

As healthcare systems strive to provide effective and efficient rehabilitation services, the availability of accurate, reliable, and comprehensive data becomes paramount [1]. This is especially crucial in environments where resources are scarce, and decision-making must be based on highly informed and strategic data. South Africa, like many low- and middle-income countries (LMICs), faces challenges in providing adequate rehabilitation services to its population [2]. The country’s quadruple burden of disease, comprising hyperendemic HIV/AIDS and tuberculosis; increased rates of injury and violence; high maternal, neonatal and child mortality; and a growing prevalence of non-communicable diseases, places immense strain on the healthcare system [3]. Thus, the burden of disability and functioning problems related to chronic health conditions is a growing concern [4]. In these low-resource contexts, primary healthcare (PHC) rehabilitation centres have emerged as a potential solution to bridge this gap in addressing the rehabilitation needs of communities, particularly the poorer underserved populations. However, most patients attending PHC in these settings cannot access the rehabilitation services they need [5]. The National Health Insurance (NHI) is being implemented in the South African healthcare sector to ensure that high-quality healthcare is accessible to all populations [6]. A national health information system (HIS) has been identified as a critical element to achieving this objective [7].

South Africa operates a two-tier health system, with a chronically underfunded public sector serving almost 80% of the population, while a privately insured sector operates parallel to this system via medical insurance, out-of-pocket payments and hospital plans [8]. Primary healthcare facilities, including PHC rehabilitation centres, are funded and governed by provincial departments of health and are expected to report routine service data through the District Health Information System (DHIS) National policies such as the District Health Management Information System (DHMIS) policy [9] and the National Digital Health Strategy [10] prioritise standardized, quality health data to support planning, performance monitoring, and strategic purchasing under the NHI reform. Continued analysis, monitoring and evaluation of data on PHC rehabilitation services is required to assess the performance of the rehabilitation sectors at this level of heath care and predict the need for further rehabilitation services [11].

One of the main sources of disability and rehabilitation data besides research data repositories (surveys and censuses) are the administrative patient health records. A significant amount of data, which can be used for routine reporting and health system management, is generated from patients’ medical records following consultations, diagnostics (e.g., laboratory and radiological tests), treatments, follow-ups. The administrative records are derived from initial bookings for rehabilitation services and case-monitoring systems often found in electronic or paper formats. These include patient registers, appointment books, therapy logs, and summary forms that capture service volumes, patient characteristics, and service types. While health institutions in the private sector have been using different e-health technologies for many years, the South African Department of Health is in the process of implementing e-health in the public sector [10]. The records can be used to generate statistics that profile new, current and past rehabilitation patients as well as patients who defaulted treatment. The data help to more accurately estimate the burden of disease including its prevalence, incidence and impact on functioning on the population entering the health system [12]. They also provide insights into the effectiveness of rehabilitation service delivery overtime and across geographical areas. Such data can be triangulated with survey data to enhance the accuracy and depth of statistical analysis, further supporting program evaluation and policy development in the field of disability and rehabilitation. However, the quality of the available data remains questionable [13].

Good data quality in PHC rehabilitation serves multiple critical functions. In low-resource settings, where data scarcity is a common challenge, the value of good quality routine data is amplified. Firstly, it enables evidence-based decision-making, allowing healthcare providers and policymakers to develop and implement effective rehabilitation policies and programs, and allocate limited resources effectively [14]. This is important in addressing the health inequities that exist in South Africa’s healthcare where rehabilitation needs are often sidelined in health service policy planning [15]. Rehabilitation does not receive adequate financial nor workforce allocation to meet the ever-evolving population’s needs [16]. For example, in one South African provincial health budget analysis, rehabilitation services received approximately 96 million ZAR (South African Rand), representing approximately 0.3% of the total provincial health budget [17]. This equated to 28 ZAR (1,68 United States Dollars) per person being earmarked for rehabilitation. Reliable data are needed to inform workforce planning and resource allocation, particularly in South Africa’s PHC settings, where only 3% of the public-sector rehabilitation workforce is employed [18]. Secondly, it facilitates the monitoring and evaluation of rehabilitation services, ensuring that the priority needs of the population are met, and desired outcomes are achieved [19]. Lastly, high-quality data supports research efforts, contributing to the growing body of knowledge in rehabilitation science and informing best practices [20]. Hence, factual representation of patient profiles (including socio-demographics, health conditions), treatment and outcomes, and trends in service utilisation (including waiting times, frequency and referral patterns) are critical for objective service, policy and financial decision-making.

The World Health Organization (WHO) has proposed core rehabilitation facility indicators, for which data are required from service providers and for which rehabilitation data quality may be measured [21]. Data quality is a critical component of data governance, the latter being a strategy for the overall management of data usability, availability, integrity, quality and security to ensure their maximum potential [22]. Data quality is a multidimensional concept, which can be perceived and conceptualised through a set of virtues or components known as data dimensions. Thus, data can be prone to variable levels of quality in terms of accuracy, completeness, consistency, timeliness, validity, uniqueness among other factors [22]. Data quality may be defined at various levels of granularity. A recent review of data quality evaluation frameworks [23] determined that most frameworks are tailored toward the domain of data creation and the use or type of data. Thus, data needs to be contextually relevant for any meaningful analysis and interpretation.

Improving data quality in these contexts is not just a matter of enhancing existing systems but often involves building data collection and management capabilities from the ground up. However, achieving good data quality in low-resource settings presents unique challenges. Limited technological infrastructure, shortage of trained personnel, and competing healthcare priorities can all impede efforts to collect and maintain high-quality data [18, 24]. Additional factors including variations in clinical practice, lack of standardised protocols for data capturing, non-intuitive electronic health record system design and copy-paste cultures contribute to low-quality data [25]. Despite these obstacles, several initiatives have demonstrated that significant improvements in data quality are possible [7], even in resource-constrained environments such as Uganda [26].

Routine rehabilitation data from South Africa’s PHC settings have not previously been systematically assessed using a standardised data quality framework. Therefore, this study aimed to analyse the availability and quality of a sample of rehabilitation routine data from a PHC rehabilitation centre in South Africa, using the WHO’s guidance on rehabilitation data quality to identify areas for improvement regarding completeness and consistency. Identifying the existing methods for collecting data, the data which is missing and understanding the patterns is crucial for next steps in developing tailored strategies for improving data quality, not only at the rehabilitation centre but also across other South African PHC rehabilitation facilities and similar LMIC settings.

## Methods

### Study design

This facility-based retrospective descriptive case study was carried out from August 2024 to February 2025 and involved a document analysis of the rehabilitation centre’s annual reports and supporting documents (registers, tally sheets and monthly summary reports) from 2014 to 2023. These sources represent the data that are used for service management, planning and reporting to higher levels of the health system, rather than raw patient files. The rehabilitation centre was pragmatically selected due to rehabilitation services being provided within a PHC setting. Furthermore, its affiliation and proximity with a rehabilitation tertiary training institution made it convenient to access the rehabilitation centres’ documents.

### Setting

The rehabilitation centre project was initiated over 30 years ago through a bilateral agreement between the National Department of Health and the rehabilitation tertiary training institution to improve healthcare access to the low socioeconomic community. The township is situated in the Western Cape province of South Africa. The 2011 census reported a population of 54 006, predominantly Afrikaans-speaking residents and mainly from the Coloured population group [27]. Further statistics from the same census indicated a 27% unemployment rate and 15% with uncompleted or no primary education [27]. The community is often affected by intermittent gang violence.

The rehabilitation centre staffs one full-time occupational therapist (OT) and one full-time physiotherapist (PT). There are part-time clinical supervisors from the rehabilitation tertiary training institution from OT and speech and language therapy (SLT). Third- and Fourth- year undergraduate rehabilitation students from the rehabilitation tertiary training institution receive training at the rehabilitation centre during clinical block rotations. During these visits they are engaged in assessment and treatment of patients, home and work visits, community assessments and outreaches, service-learning projects, and health promotion.

### Data collection

The required documents were requested and accessed through the rehabilitation centre’s manager and the rehabilitation tertiary training institution divisionary heads. The researchers explained the purpose and objectives of their study to the relevant stakeholders. The electronic documents were emailed to one of the researchers and stored on a password protected share folder for continued access by the researchers. The paper files could only be viewed at the rehabilitation centre.

A preliminary analysis of the sources of data was done to understand the data and get an overview of the available data. The data were collated in Tables in Microsoft Excel according to the rehabilitation disciplines, namely physiotherapy, occupational therapy and speech-language therapy. Data extracted included the number of rehabilitation sessions (individual treatment sessions, home visits, therapeutic and community group sessions), and the patient health conditions. No patient identifying data were extracted. It was then possible to decide on the critical data elements or reporting benchmarks and the level of completeness and consistency to measure.

### Data analysis and reporting

#### Data availability

To assess the availability of monthly and annual rehabilitation reports, we verified the presence or absence of these reports on file at rehabilitation centre. A follow-up was conducted with the rehabilitation centre’s manager and rehabilitation tertiary training institution divisionary heads regarding the data that was missing. No further data could be retrieved, and the missing records were confirmed as unavailable. The reasons provided for the missing records were documented and used to inform the assessment of data availability. We noted the formats and methods used to record the data and security of the data.

#### Data quality: completeness and consistency

The documents were analysed for quality according to an established data quality assessment framework which included two intrinsic quality dimensions: (i) completeness and (ii) consistency [28]. Column analyses, table analyses, and cross-table analyses were done. We relied on key variables for conducting rehabilitation data quality assessment recommended by WHO in their Guidance on the Analysis and Use of Routine Health Information Systems (RHIS) – Rehabilitation module (Table 1) [21]. However, timeliness was not assessed with completeness in this study because the available routine data were historical (2014–2023) and not linked to real-time reporting or submission timestamps, making valid timeliness assessment infeasible.

**Table 1:** Routine data quality assurance metrics related to completeness and consistency (from the WHO’s RHIS module [21])

Domain	Data quality metric	Method	Frequency
Completeness	Completeness of reporting (reporting form/data set completeness)	Measure whether all entities for reporting do so (compared to nine core facility indicators for rehabilitation shown in Table 2)	Monthly, annually
	Completeness of indicator data (data element completeness)	Measure whether all data elements are reported	Monthly, annually
Internal consistency	Presence of outliers	Check for any data values that are extreme in relation to other values in the series	Monthly, annually
	Consistency over time	Check whether any reported values are extreme in relation to previously reported values during the year or over the years.	Monthly, annually
	Consistency between indicators	Check whether the relationship predicted in the reported data, is as anticipated.	Annually
	Consistency between denominators, e.g. estimated number of new cases with selected health condition; estimated population at subnational level		Annually
External consistency with other data sources	Consistency between routinely-reported data and population-based surveys		Excluded (no other data sources to compare)
External comparison of population data	Consistency between the population data used for calculating rehabilitation coverages and other sources of population estimates		Excluded (no population data to compare)

* WHO RIS: World Health Organization’s Guidance on the analysis and use of routine health information systems: rehabilitation module

The module aims to standardise data on rehabilitation services and assistive technology. The data quality was assessed according to the predetermined criteria as follows:

#### Data completeness

To measure the level of data completeness, the data elements in the submitted Microsoft Excel forms were examined for completeness using two metrics: reporting form completeness and key data element completeness.

The first metric checked whether the rehabilitation centre reporting form enabled data on the applicable elements from the core rehabilitation facility indicators listed in the WHO’s Guidance on the Analysis and Use of RHIS – Rehabilitation module to be captured [21]. Table 2 indicates the nine indicators checked on (eight recommended for all types of rehabilitation facilities and one for PHC settings) and the amendments made to this checklist to align with the context of rehabilitation centre.

**Table 2:** Subset of core facility indicators for rehabilitation from the WHO’s RHIS module [21], which were used as a checklist for assessing data completeness at primary care rehabilitation centre

Core indicator	Definition	Disaggregation	Amendments to checklist used for this study
Rehabilitation personnel density	Number of rehabilitation workers/ Total population x 10 000	Rehabilitation occupational group	None
Rehabilitation uptake	Number of rehabilitation sessions provided/ Total population x 10 000	Health condition group Rehabilitation occupational group	Number of rehabilitation sessions provided/Total available sessions/month x 100
Rehabilitation service utilization	Number of cases that receive rehabilitation services/ Total population x 10 000	Health condition group Sex Age (0–4 yrs; 5–17 yrs; ≥18 yrs)	Number of cases that receive rehabilitation services/Total available sessions x 100
Assistive products uptake	Number of assistive products provided	Six categories of the WHO APL Age (0–4 yrs; 5–17 yrs; ≥18 yrs)	None
Outreach programmes uptake	Number of rehabilitation sessions provided by outreach programmes	Sex Age (0–4 yrs; 5–17 yrs; ≥18 yrs)	None
Rehabilitation referral	Number of referrals x 100/ Total number of new cases accessing the facility	Service type (assistive product provision, and other rehabilitation services)	None
Waiting time for assistive product provision	Number of days waiting for assistive product provision/ Number of assistive products provided	Six categories of the WHO APL	None
Rehabilitation waiting time	Number of waiting days until first session/ Number of new cases	Rehabilitation occupational group	None
Essential Package availability	Is facility offering an Essential Package for rehabilitation?	Essential Package type	None
Rehabilitation bed density	Number of rehabilitation beds/ Total population x 10 000.	Geographical region	Excluded (The facility does not have an inpatient or long-stay rehabilitation ward.)
Individualized care plan	Number of new inpatients receiving an individualized care plan x 100/ Number of new inpatients	Geographical region	Excluded (Without any inpatients, no care plan data are recorded)
Length of stay	Number of inpatients stay for discharged clients / Number of discharges	Health condition Geographical region	Excluded (The centre has no inpatient or long-stay ward, therefore inherently no inpatient discharges to calculate an average length of stay)
Functioning change	Difference between the average functioning assessment score at admission and at discharge	Health condition Geographical region	One health facility included so not done per geographical region
Coverage for people with acute and complex needs	Number of first-time admissions for selected health conditions x 100/ Estimated number of new cases for selected health conditions	Health condition	Excluded (The facility does not handle inpatient admissions for acute conditions and does not have access to or compile the epidemiological estimates of new cases in the catchment area)
Accessibility for people with acute and complex needs	Number of first-time admissions for selected health conditions	Health condition Geographical region	Excluded (The facility does not formally admit patients for rehabilitation after acute events and does not track how many people with acute and complex needs are able to access the centre’s services)

*WHO APL: World Health Organization’s Assistive Products Priority List; yrs: years

Each core indicator was addressed separately with its data completeness status as follows:

- Complete: all data required to compute the applicable core indicators, reported in the specified format and disaggregation
- Partially Complete: some data reported, but not in the required format or missing disaggregation
- Incomplete: no data captured/absent

The second metric involved examining the completeness of the indicator data reported by identifying gaps or missing key data elements/values in the reports through column-by-column and table analyses. A missing/null value could have occurred either because the value existed but was not known, the value did not exist, or because it was unclear whether it existed. Completeness was assessed at the level of the reporting year and data element because the annual summary reports are derived from the daily, weekly or monthly tallies of the key data elements provided in the Excel sheets. These reports should provide one annual value per discipline for each data element and were cross-checked for accuracy. Thus, the following generic formula: *Completeness = Number of not null values/total number of values* [28] was adapted to *Completeness = Number of years with a reported value/total number of years assessed (10 years)*. The key data elements selected were (i) number of rehabilitation personnel, (ii) number of rehabilitation sessions and (iii) patient pathology profiles.

#### Data consistency

The internal and external consistency of the rehabilitation centre on rehabilitation personnel, rehabilitation service utilization, assistive product uptake, length of stay and functioning change were assessed using the metrics for data consistency outlined in Table 1.

The following formula could have been used and indicated as a percentage of records submitted per data element over the 10-year period: *Consistency = Number of consistent values/number of total values* [28]. However, due to the inadequate data, the consistency was reported on narratively through a description of potential discrepancies and outliers in the data.

## Results

### Assessment of data availability

Over the ten-year period included in the analysis of data availability (2014–2023), annual reports were available at the rehabilitation centre as PDF documents for seven of the ten years (70.0%), while three years (30.0%) had no annual reports on file. The annual reports documented the centre’s client demographics, treatment statistics, and program outcomes. The reports detailed various services provided, including OT, PT and SLT, as well as the centre’s involvement in health promotion and community outreach programs. The reports highlighted the role of the university’s students in the centre’s activities through their internships and participation in service-learning projects. The students’ activities included patient assessment and treatment, home and workplace visits, community outreach, telerehabilitation and health promotion activities conducted under clinical supervision.

The monthly reports, as supporting routine documentation for the available years, were presented as Microsoft Excel sheets containing daily and weekly tallies of patients seen. These were available as separate Microsoft Excel sheets from the different rehabilitation disciplines. The available data systems were not linked to other health facilities and services to enable data sharing and knowledge exchange.

Poor security measures were evident as there were reported losses of data due to loss of files or when staff leaves.

### Assessment of completeness of the reporting form

**Fig. 1:**
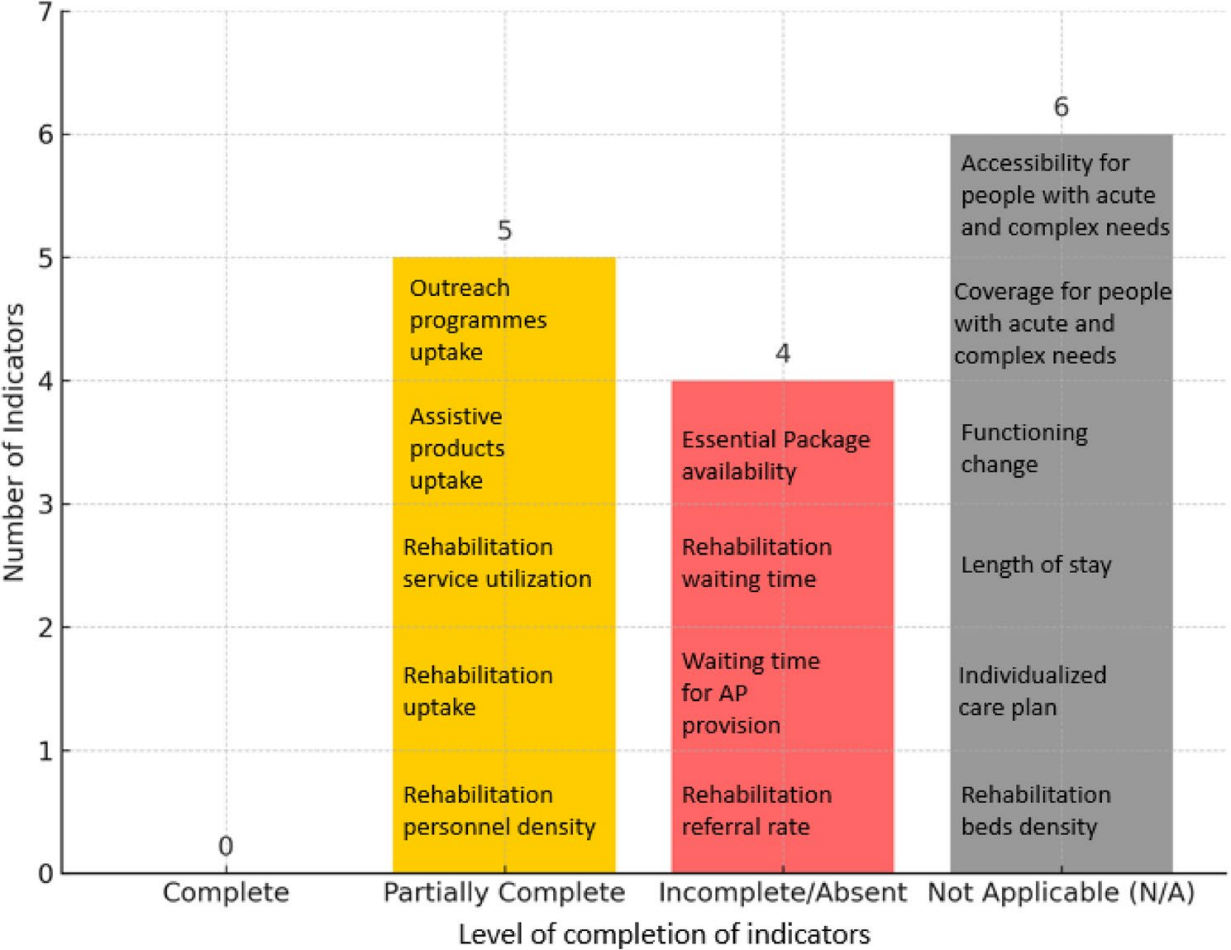
Completeness of reporting form according to rehabilitation core indicators

Nine of the 15 indicators were considered in assessing completeness of the rehabilitation centre’s data form, while six were excluded because the rehabilitation centre was an out-patient facility (Fig. 1). Overall, no indicator had fully complete data; five were partially complete (some data reported, but not in the required format or missing disaggregation) and others were incomplete (no data captured or absent due to not being captured or lack of data).

1. Rehabilitation personnel density

Status: Partially complete

Explanation: The facility recorded the number of rehabilitation workers by occupational group but did not calculate this as a population-based density. In other words, they have the counts of staff (e.g. number of PTs or OTs, etc.) but did not relate it to the population size (per 10,000 population) as the indicator definition requires.

2. Rehabilitation uptake

Status: Partially complete

Explanation: The data collected for rehabilitation uptake included the number of rehabilitation sessions provided, broken down by occupational group and health condition, but these figures were not converted into a rate. The WHO indicator requires an indication of sessions related to the population size. In the rehabilitation centre’s context, population data from the national censuses or community could be used to calculate the rate per population density. The rehabilitation uptake may be presented as a proportion of the available rehabilitation sessions at rehabilitation centre. For example, if one therapist is expected to provide eight sessions per day, this can be used to estimate the number of sessions provided at the facility.

3. Rehabilitation service utilization

Status: Partially complete

Explanation: The centre reported the number of cases that received rehabilitation services, disaggregated by health condition group, but did not calculate this as a rate per population or per available sessions. Additionally, the data was not broken down by sex or age group as required. This means two components of the indicator were missing: (i) the service-based utilization rate and (ii) the demographic disaggregation (by gender and age categories).

4. Assistive products uptake

Status: Partially complete

Explanation: Data on assistive products was collected, including the number of assistive products issued and their types. Assistive products included mobility aids (such as wheelchairs, crutches, and walking frames), upper-limb and lower-limb splints, orthoses, hearing and communication aids, and basic self-care assistive products provided to patients through the rehabilitation centre. However, the data on assistive products was not categorized according to the six WHO Assistive Products Priority List (APL) categories, namely mobility, hearing, vision, communication, cognition and self-care. Furthermore, the data was not disaggregated by age group of the recipients.

5. Outreach programmes uptake

Status: Partially complete

Explanation: The facility listed how many rehabilitation sessions were conducted per outreach initiative (including community screening activities, home visits, school-based rehabilitation visits, and volunteer and home-based carer training) but did not disaggregate these data by the sex or age of the people who received those sessions. The indicator expects the number of outreach rehabilitation sessions, disaggregated by sex and age group, so without those demographic details the data remains partially complete.

6. Rehabilitation referral rate

Status: Incomplete/absent

Explanation: The rehabilitation referral indicator was not calculated. The facility only reported the number of new cases by rehabilitation occupational group (e.g. how many new patients seen by PT, OT, etc.) but did not report the number of referrals or compute the referral rate (which should be the number of referrals as a percentage of new cases, by service type). The expected indicator is the number of referrals x 100 divided by total new cases, disaggregated by service type (assistive product provision vs. other rehab services). Since only new cases (numerator of new cases by staff group) were given and the referral aspect was omitted, the data was marked as incomplete. There could have been patients who were referred but did not attend the appointment.

7. Waiting time for assistive product provision

Status: Incomplete/absent

Explanation: No data were collected on how long patients waited to receive assistive products. The indicator is defined as the average number of days waiting for assistive product provision (from prescription/request to delivery) per number of assistive products provided. In the assessment, this information was not recorded at all – there is a gap in tracking the time interval from when an assistive product is needed to when it is issued to the patient. Without any data on waiting times, this indicator could not be computed, rendering it incomplete.

8. Rehabilitation waiting time

Status: Incomplete /absent

Explanation: No data were collected on the waiting time until the first rehabilitation session. This indicator requires measuring the number of days a new patient waits from the point of entry (registration or referral to the rehabilitation centre) to their first therapy session. As a result, the facility has no measure of how quickly new patients are seen for their initial rehabilitation session, and the indicator is entirely incomplete.

9. Essential package availability

Status: Incomplete/ absent

Explanation: The indicator asks if the facility provides a defined Essential Package for rehabilitation (and to specify the type of package). The Essential Package refers to a defined set of priority rehabilitation services that are required to meet the rehabilitation needs of the catchment population, considering the prevalent health conditions and high levels of associated functioning problems and disability. The WHO has provided the Package of Interventions for Rehabilitation, which provides guidance on the workforce needs, assistive products, equipment and consumables that are needed to deliver the essential rehabilitation interventions [29]. Countries and facilities can adapt this package when planning towards the integration of rehabilitation services into their health systems to ensure equitable access and continuity of care [29]. The assessment found no data on whether the facility is offering a standardized package of services.

Additional data was found on Functioning Change, although the WHO’s guidance excludes this for PHC facilities at which no dedicated rehabilitation wards exist. However, the data was found to be Partially Complete. The indicator “functioning change” is meant to capture the difference between average functioning assessment scores at admission and at discharge for patients. Data on functional outcomes for patients was only scantily reported, with single outcome measures documented for some conditions such as knee osteoarthritis and the Oswestry Disability Index for low back pain. There were no comprehensive pre- versus post- measures for all discharges. There was an attempt to record an outcome (e.g., a score on a functional scale for a patient), but it was not done systematically or for enough patients to calculate an average change. As a result, the data are partially complete: there is some information on patient outcomes, but it’s insufficient to properly quantify overall functional improvement.

### Assessment of completeness of key data elements

#### Assessment of completeness of data on number of rehabilitation personnel

**Fig. 2:**
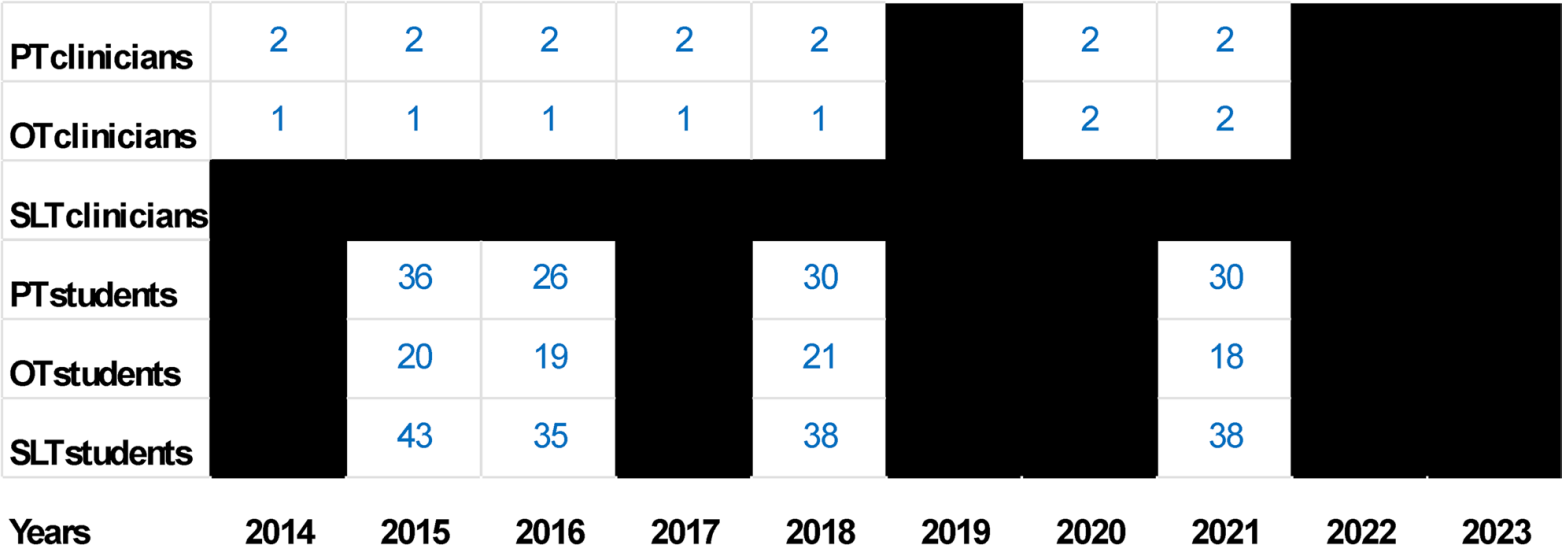
Completeness and consistency of data on the number of rehabilitation personnel, including Physiotherapy (PT), Occupational Therapy (OT), and Speech and Language Therapy (SLT) over the ten-year period. *Black regions = no data reported (thus, the percentage of clear areas correlates with the percentage of completeness,* using the year as the unit of analysis), and values in blue font are the actual/raw data values (frequency counts of rehabilitation personnel) that were assessed for consistency*

The completeness of data on the number of qualified clinicians over the ten-year period was 70.0% for both PT and OT but 0% for SLT (Fig. 2). All three rehabilitation disciplines had 40.0% completeness for data on number of undergraduate rehabilitation students who delivered patient care under clinical supervision.

#### Assessment of completeness of data on number of rehabilitation sessions

**Fig. 3:**
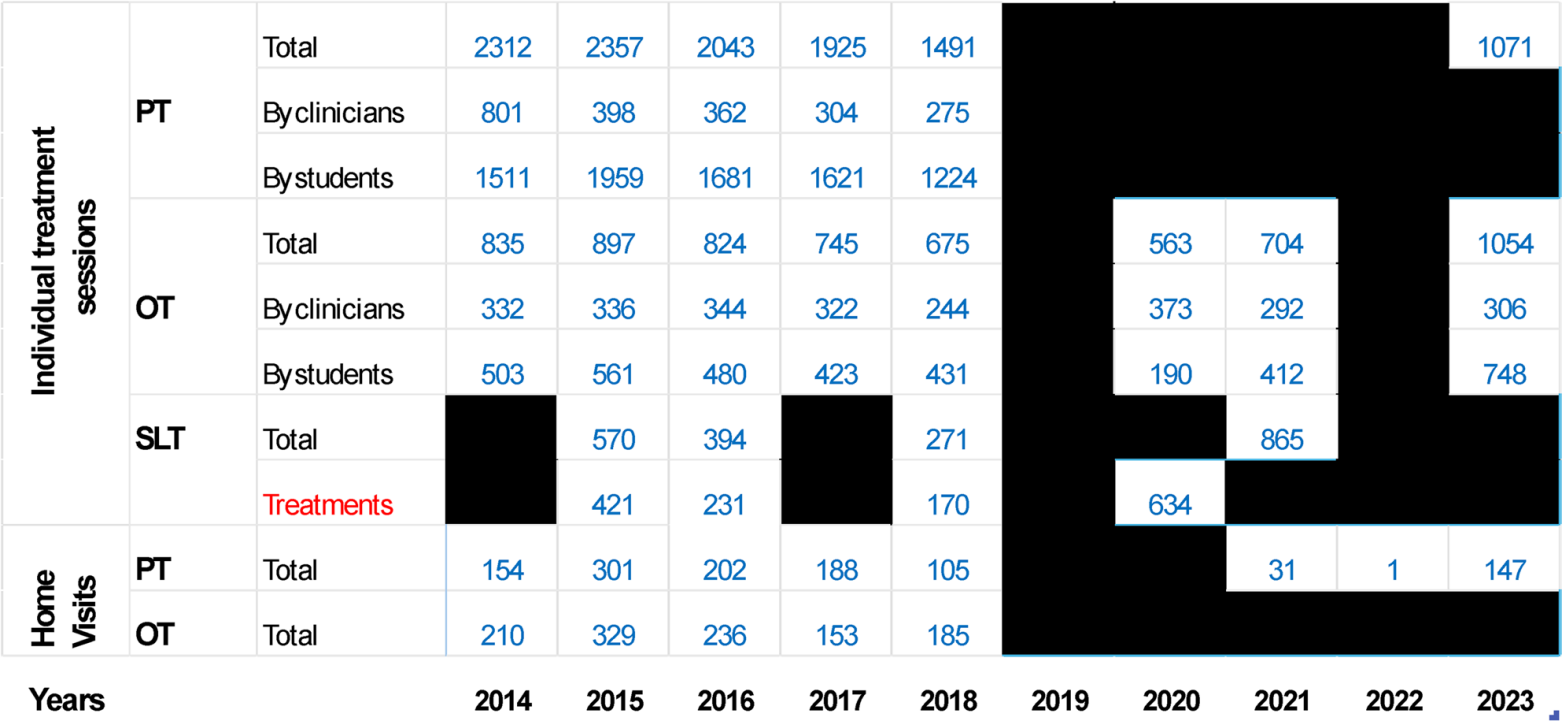
Completeness and consistency of data on individual treatment sessions and home visits from Physiotherapy (PT), Occupational Therapy (OT), and Speech and Language Therapy (SLT) over the ten-year period. *Black regions  = no data reported (thus, the percentage of clear areas correlates with the percentage of completeness, *using the year as the unit of analysis), and values in blue font are the actual/raw data values (frequency counts of individual treatment sessions and home visits) that were assessed for consistency*

The completeness of data on the number of individual treatment sessions and home visits over the ten-year period was 80.0% and 52.5% for OT and PT respectively (Fig. 3). SLT did not report any data on home visits but had 40.0% completeness for data on the number of individual treatment sessions over the ten-year period (Fig. 3). Home visits accounted for approximately 8.6% of total PT sessions and 16.8% of total OT sessions over the years for which data were available.

**Fig. 4:**
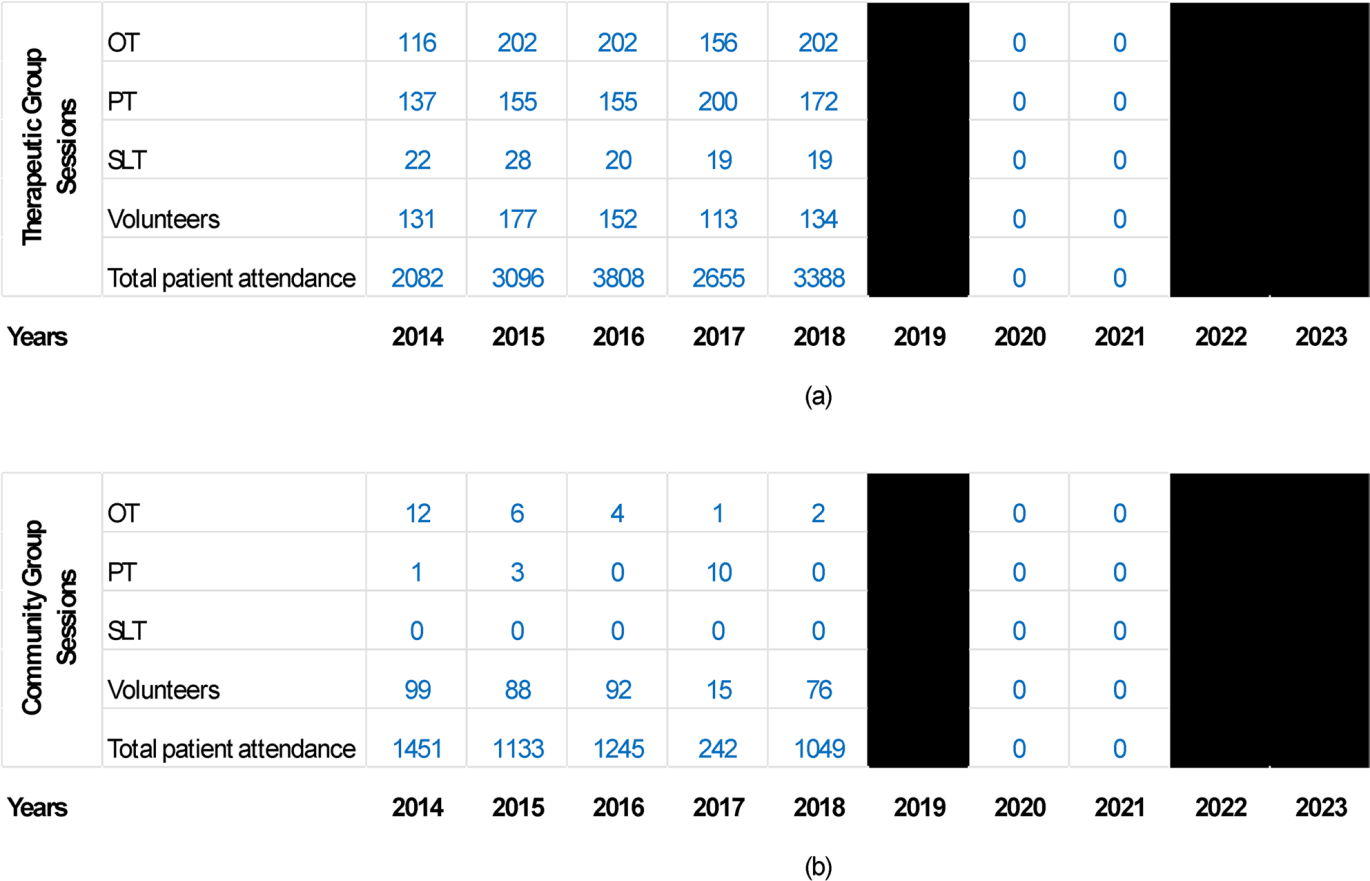
Completeness and consistency of data on (**a**) Therapeutic Group Sessions and (**b**) Community Group Sessions over the ten-year period. *Black regions = no data reported (thus, the percentage of clear areas correlates with the percentage of completeness, *using the year as the unit of analysis), and values in blue font are the actual/raw data values (frequency counts of group sessions) that were assessed for consistency*

Group session data (therapeutic and community groups) was consistently reported on from 2014 to 2018. The groups reported on included low back pain, osteoarthritis, stroke and geriatric exercise groups, and home-based care training. Data provided included number of sessions by the three rehabilitation disciplines, by volunteers, and the patient attendance. There appears to be a complete cessation of all therapeutic and community group sessions from 2019, as indicated by zeros across all rehabilitation disciplines, with no data entered for the years 2019, 2022 and 2023. Thus, this represented 70.0% completeness across all disciplines (Fig. 4).

### Assessment of completeness of data on patient pathology profiles

**Fig. 5:**
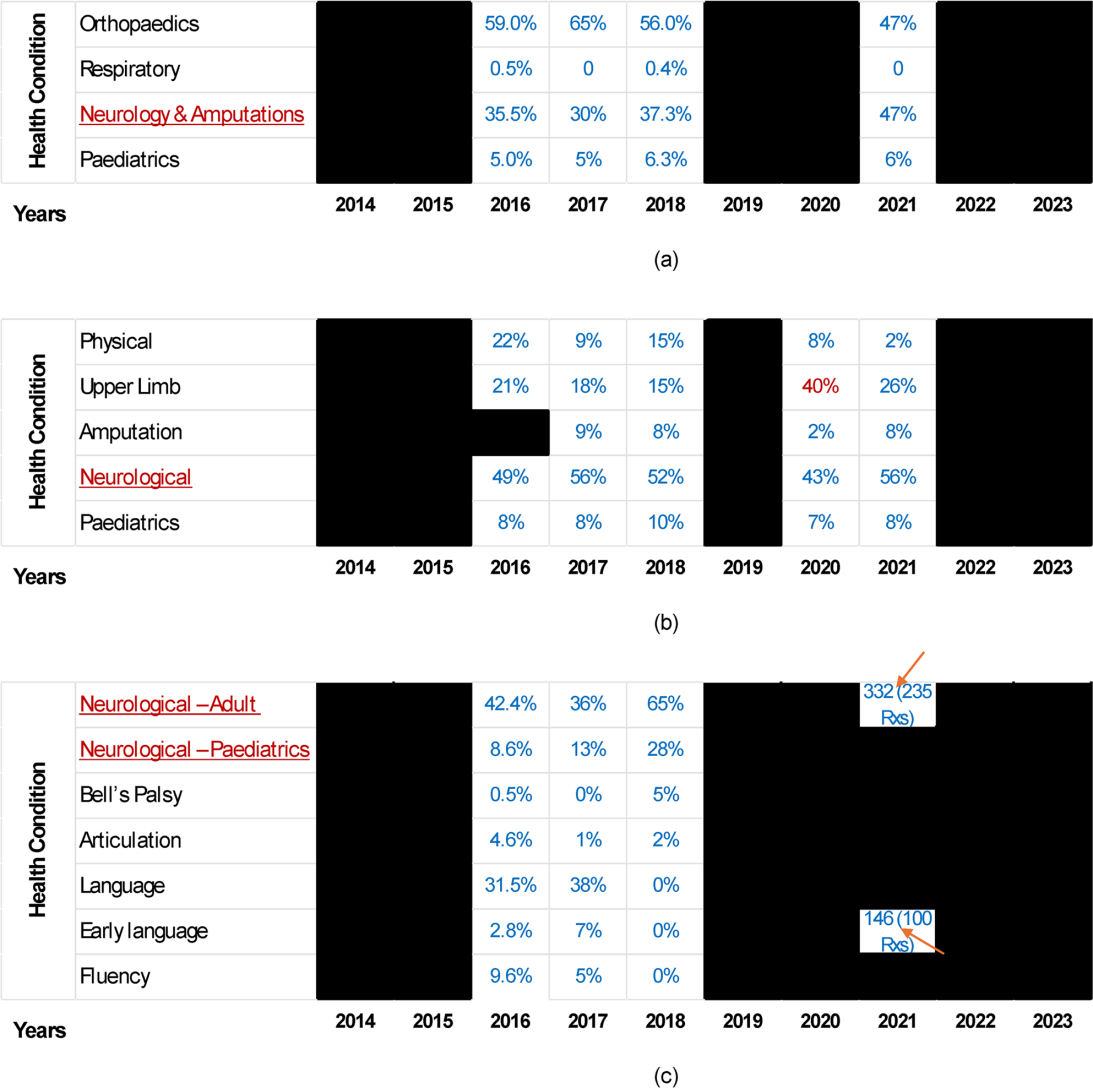
**a**-**c**: Completeness and consistency of data on health conditions and diagnoses from (**a**) Physiotherapy, (**b**) Occupational Therapy, and (**c**) Speech and Language Therapy over the ten-year period. *Black regions = no data reported (thus, the percentage of clear areas correlates with the percentage of completeness, *using the year as the unit of analysis), and values in blue font are the actual/raw data values (frequency counts of patients with a diagnosed health condition) that were assessed for consistency*

The completeness of data on the health conditions and diagnoses of patients seen by PT, OT and SLT over the ten-year period was 40.0%, 48.0% and 34.0% respectively (Fig. 5).

### Assessment of data consistency

The following data inconsistencies were noted across the disciplines:

1. Different formats for reporting data. For example, SLT used a separate reporting format in 2021 using raw case numbers instead of percentages when depicting patient pathology profiles (arrowed on Fig. 5c). Additionally, the number of decimal places used to report values was inconsistent across years and disciplines.
2. There was inconsistency in the naming and/or grouping of categories reported on. For example, PT provided combined data on Neurology & Amputations, while OT reported on these categories separately. SLT reported on two groups of Neurology (adults and paediatrics) (underlined on Fig. 5a-c). An additional example includes SLT reporting on number of treatment sessions without classifying them into treatment provided by clinicians and provided by students (underlined on Fig. 3).
3. Unexplained gaps in data. For example, while figures were reported for number of rehabilitation sessions in 2014 and 2015, none of the disciplines reported the patient pathology profiles for the same years. Conversely, PT did not report on number of rehabilitation sessions (Fig. 3) but reported on patient pathology profiles in 2021(Fig. 5a). OT is the single discipline that reported complete data on health conditions and diagnoses for the year 2020. SLT displayed the most pronounced inconsistencies with data on number of rehabilitation sessions being available for four years (2015, 2016, 2018 and 2021) but reporting on patient pathology profiles for 2016–2018 and some for 2020.
4. Unexplained fluctuations noted in the reported values. For example, the percentage of patients with Upper Limb conditions recorded by OTs in 2020 were approximately double those reported in the other years for which data were available (Fig. 5c).

However, there were consistencies noted in that, in all the years where data were reported, category percentages totalled 100%. There were consistencies in the greater proportion of treatments provided by students in comparison to clinicians over the years. There were consistent patterns in the dominant pathology groups for each discipline; orthopaedic conditions for PT, and neurological conditions for OT and SLT.

## Discussion

The analysis of record-keeping at the rehabilitation centre over the ten-year period revealed significant challenges in data quality that mirror broader issues faced by PHC facilities in low-resource settings. The findings highlighted systematic gaps in data availability, completeness and consistency that limit the facility’s ability to effectively monitor and evaluate its rehabilitation services. The study demonstrates, not only the absence of data, but where, how, and why routine rehabilitation data fail to meet WHO standards, thereby identifying concrete, system-level targets for improving rehabilitation information systems.

### Availability of data

Our study’s findings reflected the absence of an efficient HIS to collect quality rehabilitation data at the rehabilitation centre. The data were collected from the different rehabilitation divisions who submitted various Microsoft Excel sheets or paper-based registers. This mirrors findings by Gimbel et al. (2011), who identified reliance on paper-based and siloed data collection systems as a major barrier to achieving high-quality healthcare data in resource-limited settings [30]. Investing in a centralized low-burden electronic HIS would facilitate more efficient data collection and quality control. The Framework and Strategy for Disability and Rehabilitation Services in South Africa (2015–2020) has called for the integration of disability-related data into the national HIS to address the lack of rehabilitation data [31]. However, as cautioned by Gutenbrunner et al. (2018), such implementations must be carefully planned to ensure sustainability in resource-limited settings [32]. For example, data sharing agreements will need to be put into place to ensure that patient information can be shared across all services and be incorporated into a centralised local or national health data repository. Additional systems will be required to transmit reports from the PHC facility to higher levels including national databases.

There is no single best HIS for rehabilitation, but effective rehabilitation planning requires data on disability prevalence, functioning, access to services, and rehabilitation outcomes [33]. For low-resource settings with weak HIS, the recommended approach is to start with a minimum dataset of rehabilitation indicators and gradually improve the system, rather than trying to implement a comprehensive system all at once [33]. The experience of Uganda’s efforts to integrate the WHO’s RHIS – Rehabilitation module within its national electronic HIS has confirmed the importance of initially prioritising a minimum data set contextually which can be expanded to include more rehabilitation indicators [26]. The aim is to have a predominantly computer-based system which operates regionally, nationally and even internationally to decrease administrative burdens and streamline processes [33]. However, in low-resource settings, inadequate information and communication technology infrastructure development and management may hamper the shift to the use of web-based systems.

### Data quality challenges

The most prominent data quality issues identified at the rehabilitation centre included incompleteness of data. Some years had no data and could not be accounted for within the records. The incompleteness of data for some years correlated with the disruption in rehabilitation service provision by the COVID-19 pandemic and the associated national lockdown in 2020 [34] and safety in the area due to gang war violence in recent years [35]. However, the records did not account for the incomplete data. The lack of consistent longitudinal data particularly limits the potential for retrospective cohort studies, burden of disease tracking, and outcomes-based research, thereby hindering evidence generation to inform local rehabilitation strategies. A record of such gaps in data maintains professional accountability and informs relevant policy makers and service planners of gaps in service delivery. For example, the gaps in service may highlight the need for PHC rehabilitation policymakers to develop disaster preparedness strategies for continued service provision and data management [36].

The data completeness assessment for the rehabilitation centre’s core indicators revealed several areas needing improvement. Many indicators were only partially complete, often because calculations (like per-population rates) or required disaggregation (by age, sex, or category) were not performed even though raw data existed. Other indicators were entirely incomplete due to a lack of data collection or absence of the data such as no essential package of rehabilitation at the rehabilitation centre. A recent study that reviewed client records across nine PHC rehabilitation facilities in another South African province similarly reported incompleteness of data on key rehabilitation indicators such as referral sources and discharge information [37]. These findings and ours reflects a common challenge in low-resource PHC settings, where competing priorities and resource constraints often relegate data collection and management to a lower priority [38]. This gap may impede efforts to improve continuity of care and co-ordination with other levels of healthcare. To advance toward complete data for all core indicators, the centre could implement the authors’ recommendations provided for each specific measure (Table 3). Common themes include integrating simple calculations into routine reporting (for rates and percentages), expanding data collection forms to capture necessary details (such as demographics or dates for waiting times), adopting standard definitions and categories (like WHO-APL for assistive products). By systematically addressing the gaps for each indicator, the rehabilitation centre can achieve more complete and informative reporting.

**Table 3:** Recommendations to improve completeness of data in alignment with WHO’s guidance [21]

Core indicator	Recommendation
Rehabilitation personnel density	Integrate workforce data with population statistics to calculate rehabilitation personnel density.
Rehabilitation uptake	Use the formula: Rehabilitation Uptake=Total Rehabilitation Sessions/Total Population×10 000 Ensure that data collection tools capture total population figures to enable accurate calculations.
Rehabilitation service utilization	Implement electronic patient records that include demographic fields (age, sex). Structure reports to categorize service utilization by sex, age group (0–4 yrs, 5–17 yrs, ≥ 18 yrs), and health condition.
Assistive products uptake	Align data collection tools with WHO-APL categories (six priority types). Disaggregate data by age group to assess equitable distribution. Implement inventory tracking systems to automate categorization and reporting.
Outreach programmes uptake	Update data collection templates to include sex and age categories. Ensure outreach teams record demographic data systematically for reporting consistency.
Rehabilitation referral	Modify referral forms to capture service type. Develop an integrated referral tracking system to monitor patient pathways from referral to service provision.
Waiting time for assistive product provision	Introduce a timestamp system at the point of prescription and product issuance. Calculate waiting time using: Waiting Time = ∑ (Date of Issuance − Date of Prescription)/ Total Assistive Products Provided
Rehabilitation waiting time	Implement a waiting list management system to record the date of referral and the first session date.
Essential Package availability	Define and document the Essential Package for rehabilitation at the facility.

*WHO APL: World Health Organization’s Assistive Products Priority List; yrs: years; ∑: summation of

The review also identified inconsistencies in reporting data, particularly between the rehabilitation disciplines. This made it difficult to compare data across the disciplines and over the years. A comparative study of nine middle-income countries using WHO’s Systematic Assessment of Rehabilitation Situation (STARS) found that although health facilities in all countries collected service utilization data, in seven countries this was neither standardised nor collated [39]. Agreement on quality indicators and data points, along with well-developed definitions, is required to ensure harmonisation [13, 40]. In South Africa, this may be done under the guide of the National Department of Health to prevent ad-hoc and uncoordinated implementation of new indicators. A first step is to determine the information required in the current context to develop standardised core data elements to enable comparisons and benchmarking. Developing a standardized data collection tool aligned with WHO’s core rehabilitation facility indicators would improve data consistency and completeness [21]. The tool should provide national definitions for the extraction of data on rehabilitation patients including personal and demographic, social and personal circumstances, care events and screening activity, referral, diagnoses of health conditions and functioning problems/disabilities, and outcome measures. Standardisation will reduce unwanted variation when sharing/communicating data across the healthcare system and improve the usability of the data [21]. The same tool can be used as a standard benchmark for auditing data quality in PHC rehabilitation settings.

However, the current study identified positive aspects on the data collected. Though not having a dedicated rehabilitation ward, the rehabilitation centre was able to have a partially complete report on some core rehabilitation indicators such as functioning change. This highlights that it is possible to consider reporting on additional elements beyond those that the WHO’s RHIS – Rehabilitation module recommends for PHC facilities. The adoption of the International Classification of Functioning, Disability and Health (ICF) framework could provide standardised descriptions of functioning and disability problems in the rehabilitation centre’s HIS [41]. Thus, it may be possible to capture data on specific functioning or disability problems, such as pain, mobility and mental functioning-related problems, in addition to the current profiling of patients according to their health conditions or diagnoses. This rehabilitation-specific data are important in determining population rehabilitation needs and guiding service development and resource allocation [1]. Rehabilitation-specific performance indicators will help determine whether the specific rehabilitation services provide optimal or adequate patient care [40]. Thus, it will be useful for the rehabilitation centre to implement standardized functional assessment tools aligned with the ICF (e.g., WHO Disability Assessment Schedule [WHODAS] 2.0) to collect pre- and post-intervention scores to assess rehabilitation effectiveness.

Our findings demonstrate where the current rehabilitation routine data fall short within the available WHO’s routine data quality monitoring framework, with clear benchmarks and indicators to identify and address quality issues promptly. This aligns with WHO recommendations for strengthening HIS in PHC settings [21]. Implementation can occur through regular local and national audits/monitoring of data quality in PHC rehabilitation settings. The District HIS National policy provides standards for data governance, but these need to be adapted to rehabilitation settings [9]. Additionally, a self-assessment ‘System Conformance Checklist’ tool can be provided for providers of PHC rehabilitation services to carry out ‘data mapping exercises’ to determine how their existing electronic systems align with the benchmark standards before submitting the data [42]. Many electronic HIS have built-in tools for data quality checks. Reports from these audits can be made available for facilities to consider opportunities for improving data quality.

The quality of data presented depends greatly on the quality of the data submitted. Of the many studies that have found routine health data to be of poor quality, an underlying cause is the lack of significant value that the clinicians assign to the quality of administrative data collection [38]. To motivate improvements in submission of consistent and accurate data, measurement of data quality may be added into key performance indicators for the PHC rehabilitation facilities. Previous studies have shown that improving staff capacity in data management can significantly enhance data quality [43]. Thus, regular training for staff on data collection and quality assurance procedures may be essential.

### Next steps and recommendations

The research team will engage data users and producers in designing the pilot electronic HIS. It may be important to conduct a stakeholder analysis of health-related rehabilitation service providers or users of data for rehabilitation service planning. The end users will be useful in describing their information needs while providers can help assess the current data collection strengths, weaknesses and gaps, benefits and capacities of the current system.

Thus, future research could focus on:

- Developing context-appropriate tools for data quality assessment in rehabilitation services.
- Examining the feasibility of implementing integrated electronic HIS for rehabilitation in similar settings.
- Investigating the impact of improved rehabilitation data quality on service delivery and patient outcomes.

Future studies may extend the study to various PHC rehabilitation settings in the country and similar settings in other LMICs.

### Strengths and limitations

This study was conducted at one PHC setting and some of the challenges experienced may be unique to this setting. These include the rehabilitation centre’s strong reliance on undergraduate student clinicians, periodic staff turnover, and intermittent service disruptions due to local gang violence, all of which likely contributed to gaps, inconsistencies, and loss of records. Other limitations are likely to be shared by many PHC rehabilitation centres in South Africa and other LMICs, including paper-based or fragmented electronic systems, lack of standardised rehabilitation data collection tools, limited data management capacity, and the absence of rehabilitation indicators within national health information systems. However, the current study provides guidance for assessment of rehabilitation data quality at PHC settings, which often feed into higher tier databases, such as district and national databases.

Our findings are solely dependent on the documentation provided and the interpretation of the authors thereof. The study may have been strengthened through additional research methods such as qualitative interviews.

## Conclusion

The review of record-keeping at rehabilitation centre over the ten years indicated incomplete and inconsistent data on patient and rehabilitation services provided. The exercise provided a diagnostic picture of where the current data falls short of the WHO’s guidance on data quality. Evidence-based strategies that could address the identified data quality issues from our study include development and implementation of standardized data collection tools, national and facility-level investment in rehabilitation-specific HIS that incorporate standardized diagnostic codes (e.g., ICF-based reporting), quality monitoring frameworks and capacity-building for data entry personnel.

## Data Availability

The datasets generated and/or analysed during the current study are available from the corresponding author upon reasonable request.

## Abbreviations

HIS: Health Information System(s)
ICF: International Classification of Functioning, Disability and Health
OT: Occupational Therapy
PHC: Primary Healthcare
PT: Physiotherapy
SLT: Speech and Language Therapy
WHO: World Health Organization
